# Heart to spine measurements to detect left atrial enlargement in dogs with mitral insufficiency

**DOI:** 10.1186/s13620-019-0152-6

**Published:** 2019-11-20

**Authors:** Xavier Sánchez Salguero, David Prandi, Francisco Llabrés-Díaz, Edgar G. Manzanilla, Llorenç Badiella, Claudio Bussadori

**Affiliations:** 1 Animal Science Department, Escola Tècnica Superior d’Enginyeria Agrària, Universitat de Lleida, Lleida, Spain; 2 Betulia Veterinary Clinic, Barcelona, Spain; 3 Universitat Autònoma de Barcelona, Bellaterra, Barcelona, Spain; 4 Royal Veterinary College, Hawkshead Lane, Hatfield, Hertfordshire UK; 5 Animal and Grassland Research Centre, Teagasc, Moorepark, Fermoy, Republic of Ireland; 6 School of Veterinary Medicine, University College Dublin, Belfield, Republic of Ireland; 7 Servei d’Estadística Aplicada, Universitat Autònoma de Barcelona, Bellaterra, Barcelona, Spain; 8 Clínica Veterinaria Gran Sasso, Milano, Italy

**Keywords:** Dog, Left atrial size, Mitral valve disease, Radiographic measurement, RLAD

## Abstract

**Background:**

Radiography is useful to determine left atrial (LA) size when echocardiography is not available. Recently, the authors have described Radiographic Left Atrial Dimension (RLAD) as a new radiographic measurement to assess LA size. The objective of this study was to assess the clinical usefulness of 2 new radiographic measurements to detect and quantify left atrial enlargement (LAE) compared to RLAD and using left atrium to aortic root (LA/Ao) ratio as gold standard. These new measurements, bronchus-to-spine (Br-Spine) and RLAD-to-spine (RLAD-Spine) may be more precise in cases were LA boundaries are not well defined. Fifty dogs, 25 with and 25 without LAE were recruited. Reference LA/Ao ratio was assessed by 2D echocardiography and LAE was considered if LA/Ao > 1.6. Br-spine was measured as a straight vertical line from the main stem bronchus to the ventral border of the vertebra situated immediately dorsal to the heart base. RLAD-Spine was measured from RLAD endpoint perpendicularly to spine. The correlation of RLAD, Br-Spine and RLAD-Spine methods with LA/Ao and their sensitivity and specificity for detecting LAE were calculated. Receiver Operating Characteristic (ROC) curves were used to estimate the optimal cut-off for each method.

**Results:**

Correlations between Br-Spine, RLAD-Spine, RLAD and LA/Ao ratio were − 0.66, − 0.76 and 0.89 respectively (*P* < 0.001). Sensitivity at the optimal cut-off values for detecting LAE were 32.0, 64.0 and 96.0%, respectively. Specificity was 96.0% in all cases.

**Conclusion:**

Br-Spine and RLAD-Spine were less sensitive radiographic measurements than RLAD in detecting LAE in dogs. Both Br-Spine and RLAD-Spine may not be good alternatives to RLAD.

## Background

Several echocardiographic methods have been published to measure left atrial (LA) dimension [1–4] and the most commonly used is the left atrium-to-aorta ratio (LA/Ao) [1, 4, 5]. However, echocardiography is technically challenging and not universally available. Radiography is a simple and useful tool to detect left atrial enlargement (LAE) [6–9]; it is available virtually to all veterinary clinicians and offers additional information such as presence of pulmonary oedema. The most frequently used radiographic sign of LAE is a bulging soft tissue opacity located dorso-caudally to the carina on the latero-lateral view [10]. A new measurement to evaluate radiographic left atrial dimension (RLAD) has been recently described [9]. RLAD demonstrated good correlation with LA/Ao ratio as well as high sensitivity and specificity to detect LAE [9] in dogs affected by mitral insufficiency.

Identifying the exact location of the LA roof, which is needed for RLAD, is straightforward in cases with moderate to severe LAE. However, it is difficult in some normal dogs as left and right main stem bronchi, main pulmonary veins and the bronchial and vascular trees are superimposed to each other in this area [11]. New radiographic measurements of LAE free of this superimposition should be explored. In this study we hypothesized that bronchus-to-spine (Br-Spine) measurement could help in evaluating heart enlargement and measuring the distance between RLAD endpoint and the neighbouring vertebrae (RLAD-Spine) could help in evaluating the presence of LAE. Thus, the objective of the study was to test the clinical value of these two radiographic measurements in cases with LAE using LA/Ao ratio as a reference.

## Methods

A total of 50 client-owned dogs were prospectively included in this study in 2 groups: Group A (control group) composed of 25 healthy dogs without LAE and Group B, composed of 25 dogs diagnosed with mitral insufficiency and LAE. Dogs were assigned to one of two groups according to the absence (Group A) or presence (Group B) of LAE defined as La/Ao ratio > 1.6 [4, 6, 8]. All dogs had a complete radiographic and echocardiographic examination, complete clinical evaluation including physical exam, complete blood count and biochemistry panel within the same day. Normal dogs were those considered so according to the history, physical, radiographic and echocardiographic examinations. A mitral valve disease (MVD) diagnosis was based on the presence of a typical systolic mitral regurgitation murmur and alterations on the echocardiographic examination (LA and left ventricular enlargement, diffuse nodular thickening of the mitral leaflets and Doppler evidence of disturbed flow within the LA during systole). Any dog with cardiac disease either with a sole diagnosis different from MVD or any disease complex including MVD and another condition were excluded from the study. Dogs with pulmonary hypertension or radiographic changes compatible with left sided congestive failure were also excluded.

All radiographic measurements were obtained by the same observer (XS) using Microsoft Office Power point 2007 (Microsoft Corporation, USA) in a digital latero-lateral thoracic radiograph with the dog in right lateral recumbency. The observer was blinded to all patient details and all measurements were performed in a randomised fashion.

**Fig. 1:**
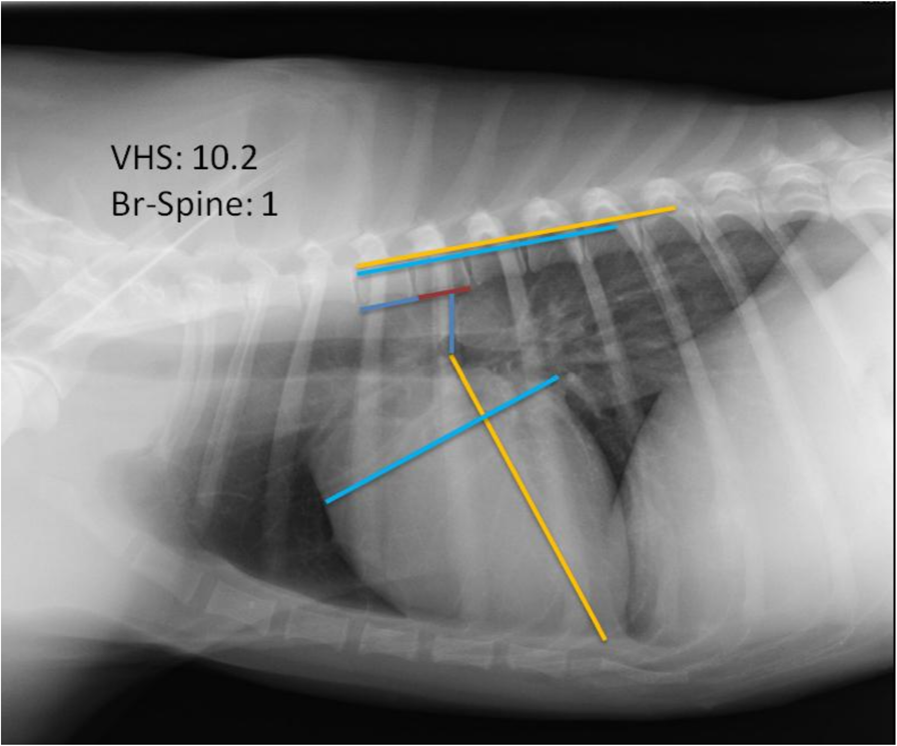
Right lateral thoracic view. The vertebral Heart Size (VHS), long (L) and short (S) cardiac axes are shown. VHS is expressed as total units of vertebral length: 10.2v in this particular case. Bronchus-to-spine measurement (dark blue line) and the distance between the cranio-ventral and caudo-ventral surface of the vertebrae dorsal to the heart base (red line) were shown. (L), (S) and Br-Spine measurements were repositioned parallel to the long axis of the thoracic vertebrae from the cranial edge of the fourth thoracic vertebra (T4). Br-Spine was thus expressed as total units of vertebral length: 1.0v

The Br-Spine was measured as the distance measured in a straight vertical line from the ventral border of the left cranial lung lobe bronchus (main stem bronchus), from the same location as VHS (vertebral heart size), to the ventral border of the vertebra situated immediately dorsal to the heart base. The dorsal reference point was defined as the intersection point between the vertical measurement from the heart base and an imaginary line between the cranio-ventral and caudo-ventral surface of the vertebrae dorsal to the heart base [5], usually T5 (Fig. 1).

**Fig. 2:**
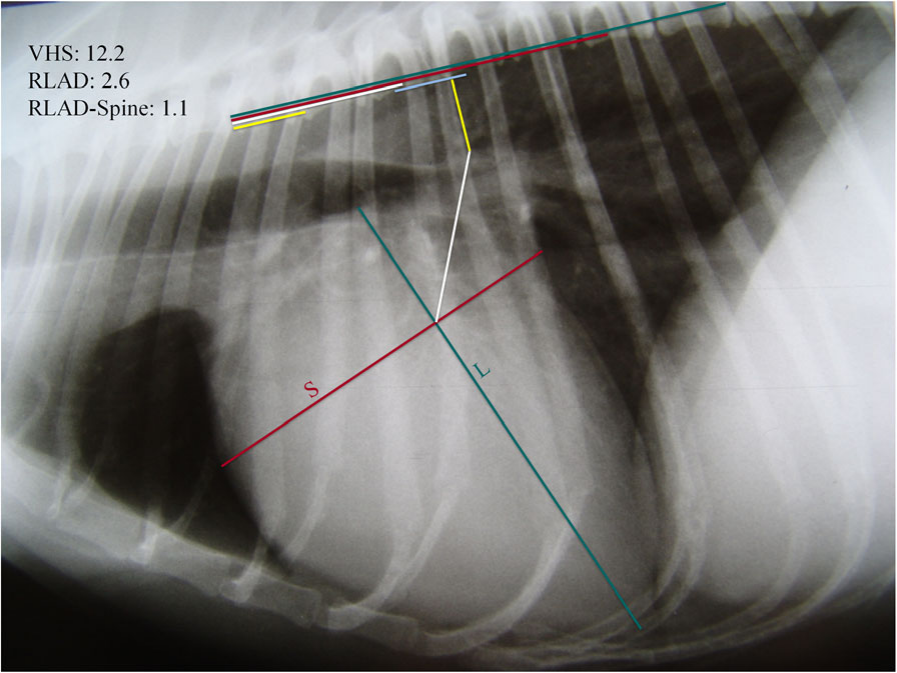
Right lateral thoracic radiograph. Vertebral Heart Size (VHS), long (L) and short (S) cardiac axes are shown. VHS is expressed as total units of vertebral length: 12.2v. Radiographic left atrial dimension (RLAD) is shown (white line). RLAD to spine measurement (yellow line) and the distance between the cranio-ventral and caudo-ventral surface of the vertebrae dorsal to the heart base (blue line) are shown. (L), (S) and RLAD-Spine were repositioned parallel to the long axis of the thoracic vertebrae from the cranial edge of the fourth thoracic vertebra (T4). RLAD-Spine was thus expressed as total units of vertebral length: 1.1v in this case

The RLAD was measured as previously described [9]. The RLAD-Spine was measured perpendicularly to the vertebrae in a straight line from the endpoint of RLAD to the ventral aspect of the vertebral column. The dorsal reference point was defined as the intersection point between the vertical measurement from the left atrial roof and an imaginary line between the cranio-ventral and caudo-ventral surface of the vertebrae dorsal to LA, usually T6 (Fig. 2). Br-Spine, RLAD and RLAD-Spine measurements were repositioned parallel to the long axis of the thoracic vertebrae from the cranial edge of T4 and expressed as total units of vertebral length to the nearest 0.1 vertebra (v). For consistency, a value of one included the length of a vertebral body and the width of the caudally positioned intervertebral disc.

A complete transthoracic echocardiographic examination was performed in all dogs by the same board-certified veterinary cardiologist according to the American Society of Echocardiography standards and guidelines and other published recommendations [12] as described in earlier reports [9].

Statistical analysis was performed using SAS 9.4 (Cary, NC, USA). Normality of data was tested using Shapiro Wilk test and visual methods. Means between groups were compared using t-test. The association between measurements was obtained by means of Spearman’s correlation. The accuracy of each test was measured by means of empirical Receiver Operating Characteristic (ROC) curve, the area under the curve (AUC) and their confidence intervals. AUC curves were compared using Delong’s method. The optimal cut-off value that jointly maximized sensitivity and specificity for each test was determined using the Youden index. Sensitivity and specificity and their confidence intervals were computed at the optimal cut-off point. Alpha level for determination of significance was 0.05.

## Results and discussion

A total of 50 dogs were prospectively included in this study, 25 dogs in the group of healthy dogs (18 males, 7 females; aged 1–7 years; weighing 4–20 kg) and 25 dogs in the group with LAE (14 males, 11 females; aged 6–10 years; weighing 4.8–21.4 kg). Breeds included mainly mixed breed dogs (*n* = 46) and pure breeds included: French Bulldog, Pug, Cavalier King Charles Spaniel and Pekingese (*n* = 1 each). Group B showed greater values (mean ± SD) for RLAD (A = 1.4 ± 0.23, B = 2.62 ± 0.55) and LA/Ao (A = 1.33 ± 0.13, B = 2.52 ± 0.56) and lower for Br-Spine (A = 1.58 ± 0.32, B = 1.10 ± 0.34), RLAD-Spine (A = 1.44 ± 0.35, B = 0.57 ± 0.48; *P* < 0.05 in all cases).

Correlation between RLAD and LA/Ao ratio was good (*r* = 0.89; *P* < 0.001) as shown in a previous study [9]. For Br-Spine and RLAD-Spine the correlation coefficient with LA/Ao ratio (*r* = − 0.66 and − 0.76 respectively; *P* < 0.001) was lower than for RLAD. Br-Spine could become a clinically relevant alternative when RLAD is difficult to measure, however, based on this data it should not be used as diagnostic index in a general population.

The ROC curves showed that RLAD optimal cut-off value was 1.8v, whereas Br-Spine and RLAD-Spine optimal cut-off values were 1.0v and 0.8v, respectively. Thus, using these reference values could decrease the overlap between normal dogs and dogs with LAE. Sensitivity and specificity were calculated based on these optimal cut-off values. Sensitivity to detect LAE was higher for RLAD (96%) than for Br-Spine (32%) and RLAD-Spine (64%). A possible explanation could be a greater effect that the dog’s thoracic conformation could have on these 2 new radiographic measurements when compared heart-based measures like RLAD. In addition, several technical factors can affect these 2 new radiographic measurements; the respiratory phase, variations in the conformation of individual dogs of the same breed and slight inconsistencies in positioning for radiography can all decrease or increase the heart-to-spine distance. Both, RLAD, Br-Spine and RLAD-Spine showed the same specificity to detect LAE (96%). These results would likely not be so high in a clinical population of dogs with causes of cardiomegaly other than left atrial enlargement.

Although differences between AUC for the different measurements were not significant, the AUCs were 0.85 (95% CI = 0.744, 0.957) for Br-Spine, 0.91 (95% CI = 0.834, 0.995) for RLAD-Spine and 0.99 (95% CI = 0.954, 1.000) for RLAD. This higher AUC for RLAD indicates a superior diagnostic value compared to Br-Spine and RLAD-spine.

The number of patients, the applicability of the methods on different thoracic conformations and the exclusion of dogs with congestive heart failure are the main limitations of this study.

## Conclusion

Br-Spine and RLAD-Spine, new radiographic measurements, are less sensitive than RLAD in detecting LAE in dogs. When LA boundaries are radiographically well visualized (in most cases, especially in dogs with moderate or severe LAE) we still propose RLAD as the best method of detecting and quantifying LAE based on its high sensitivity and sensibility in detecting LAE, as well as its high correlation with LA/Ao ratio and high AUC.

## Data Availability

The datasets used and/or analysed during the current study available from the corresponding author on reasonable request.

## Abbreviations

AUC: Area under the curve
Br-Spine: Bronchus-to-spine
LA: Left atrial
LA/Ao: Left atrium-to-aorta ratio
LAE: Left atrial enlargement
MVD: Mitral valve disease
RLAD: Radiographic left atrial dimension
RLAD-Spine: RLAD-to-spine
ROC: Receiver operating characteristic
v: Vertebrae (unit of length)
VHS: Vertebral heart size
